# Subjective Rank of the Competition as a Factor Differentiating Between the Affective States of Swimmers and Their Sport Performance

**DOI:** 10.3389/fpsyg.2020.615746

**Published:** 2020-12-23

**Authors:** Aleksandra Samełko, Monika Guszkowska, Anna Kuk

**Affiliations:** ^1^Department of Pedagogy and Psychology of Physical Culture, Józef Piłsudski University of Physical Education in Warsaw, Warsaw, Poland; ^2^Department of Occupational Therapy, Józef Piłsudski University of Physical Education in Warsaw, Warsaw, Poland

**Keywords:** athletes, emotions, mood, stress, performance

## Abstract

**Purpose:**

The aim of the study was to establish the differences in affective states of swimmers depending on the subjective rank of the competition and the relationship between affective states and performance in sports competitions of low, medium and high subjectively perceived rank.

**Methods:**

The respondents (*n* = 31) aged from 15 to 23 years (18.1 ± 2.397) were studied using the psychological questionnaires *Perceived Stress Scale* (PSS-10), *Profile of Mood State* (POMS), and *Positive and Negative Affect Schedule* (PANAS) during sports events. 362 measurements using *POMS* and 232 measurements using *PANAS* before the starts were collected. The significance of intergroup differences was determined using the Kruskal-Wallis test. A stepwise regression analysis was used to determine the emotional predictors of sports results.

**Results:**

Subjective rank of sports competition differentiated significantly anger (*chi*^2^ = 6.826; *p* = 0.033), confusion (*chi*^2^ = 11.345; *p* = 0.003), depression (*chi*^2^ = 10.2; *p* = 0.006), fatigue (*chi*^2^ = 49.394*; p* ≤ 0.001), vigour (*chi*^2^ = 11.345; *p* ≤ 0.001), positive emotions (*chi^2^* = 51.233; *p* ≤ 0.001), and negative emotions (*chi^2^* = 11.552; *p* = 0.003). Regression analysis showed the influence of mood states and positive emotions on the sports result.

**Conclusion:**

The swimmers’ affective state changed depending on the subjective rank of the competition. Depression and positive emotional state made it possible to predict the result in medium- and high-rank competition.

## Introduction

In the search for predictors of efficiency and effectiveness of sport performance, mental aspects are more and more often noticed since in combination with motor skills, they determine sports success (Cogan, 2019). In terms of mental preparation in swimming it is important to strive for an appropriate and reproducible mental state that will ensure the effective performance of a familiar task. Its important element is affective states, which may have positive or negative valence and could influence specific perception strategies (Bless and Burger, 2017).

Affective states are understood as bipolar constructs currently experienced by an individual in response to a direct or indirect interpretation or appraisal of events and stimuli. It is the combination of emotions and moods (Kontaris et al., 2020). Emotion is a more intense mental state than mood, related to physiological responses. It has a shorter duration and is usually triggered by a specific stimulus (Domínguez-Jiménez et al., 2020). Emotions and moods also differ in intentionality, causes, consequences and function. There are many models that attempt to explain the occurrence of pre-competitive emotions (Lane et al., 2012).

In the traditional approach proposed in the iceberg profile by Morgan (1980), links were sought between the negative or positive affective states and the efficiency of an athlete’s performance. This model is still used as a theoretical basis in research (Terry and Parsons-Smith, 2019; Han et al., 2020). The results of some more recent studies support the Morgan’s findings that experiencing strong positive states with low levels of negative states is conducive to the achievement of high sporting performance and that successful sportsmen and sportswomen experiencing emotions derive energy from both positive and negative emotions (Martinent et al., 2013).

The results of other studies suggest that in order to act effectively, the athlete should provide himself with as much mental energy as possible, acquired from both positive (e.g., joy) and negative (e.g., anger) affective states (Carter and Sachs, 2012). The complex relations between cognitive antecedents and mediators of affective states in sport are also of interest to researchers (Chadha et al., 2019).

Studies have demonstrated the relationships between sport performance and the appraisal of the emotional state rather than the intensity of affective states alone (Psychountaki and Zervas, 2000). Athletes who positively assessed their somatic excitement before taking part in competitions achieved better results than others (Borek-Chudek, 2011).

According to the transactional concept of stress by Lazarus and Folkman (1984), the response to a situation, e.g., a sports competition, depends on cognitive assessment. An athlete may cognitively assess the start of the competition as a threat or challenge. Lepine et al. (2005) argue that the level of stimulation alone is not as important as the individual’s perception of the task. If an athlete approaches the difficulty as a challenge, the probability of experiencing positive states increases (Gross and Thompson, 2007). In turn, conscious experiencing positive emotions improves the effectiveness of coping with stress (Tugade et al., 2004). In a competitive situation, stress depends on the situational context, including the rank of the competition (Gracz and Sankowski, 2007). The high rank of the competition can significantly modify the emotional state of the athlete and influence his or her sports result (Bittner et al., 2005).

The intensification of affective states in a sports competition may, therefore, depend not only on the objective rank of the competition (e.g., national championships), but also on its perception and subjective appraisal by an athlete: the significance of a specific competition for a specific athlete and his or her aspirations (Boldizsár et al., 2016).

Participation in the competition which is the most important from a social standpoint is not always the most important for the athlete. To date, studies on the relationships between the rank of competitions and sport performance have most often used objective rank indicators (Jeon, 2019). Assuming that the measurement of affective states before a sports performance is related to sports results, it is worth analyzing the factors influencing the experience of mood and emotions (Patrícia et al., 2019). We will attempt to fill this gap. It cannot be excluded that subjective rank of competition (understood as a sense of start importance of the athletes themselves) modifies the relationship between the affective states and the sport performance (Ihalainen et al., 2017). The subjective rank of the competition may be determined by the competitor’s performance aspirations and his or her inner motivations.

The aim of the study was to establish the differences in affective states of swimmers depending on the subjective rank of the competition and the relationship between affective states and performance in sports competitions of low, medium and high subjectively perceived rank.

## Materials and Methods

The study examined 31 swimmers at the national (Polish) level aged 15 to 23 years (18.1 ± 2.397). All participants gave written consent to participate in the study as required by the Helsinki Declaration.

In order to appraise the mood state, the *Profile of Mood State (POMS*) (McNair et al., 1971) was used, with the Polish adaptation of Dudek and Koniarek (1987). The psychometric properties of the inventory are satisfactory (Cronbach’s alpha is in the range 0.74–0.91). The tool has been used many times in the studies of athletes’ mood states, starting with Morgan’s research (1980). The adjectives form six scales: depression, tension, anger, fatigue, confusion and vigor. The Polish version of POMS contains an additional scale of kindness and has satisfactory psychometric properties (Dudek and Koniarek, 1987).

*Positive and Negative Affect Schedule* (PANAS) (Watson et al., 1998) with (Brzozowski, 2010) Polish adaptation (2010) was used to study emotional states. The tool contains the scale of positive and negative affect. The reliability of the scale is satisfactory: the Cronbach’s alpha ranges from 0.73 to 0.95 depending on the version and type of sample. The validity of the scale is confirmed by the results of factor analysis, cluster analysis, correlations with other tools and intergroup differences.

The *Perceived Stress Scale (PSS-10)* was used to determine the intensity of stress in the period before the competition. The authors of the original version of the test are S. Cohen et al. (1983), whereas adaptation to the Polish conditions was prepared by Juczyński and Ogińska-Bulik (2009). The validity and reliability of the Polish version of the test are sufficient for the purposes of scientific research.

### Procedure

The respondents completed the questionnaires in writing. Before the study began, they had received detailed instructions from one of the researchers – a psychologist. Completion of the questionnaires was supervised by three trained assistants.

The actual stage of the study took place during a sports competition. Each sports competition lasted from 3 to 5 days and had two sessions during 1 day. The swimmers were asked to fill in the PSS and POMS during the general warm-up (approx. 1 h before the first start in the morning or afternoon session) in the swimming pool halls.

Moreover, just before (approx. 10 min) each start (race), the swimmers filled out the PANAS questionnaire. Throughout the competition, the assistants observed the athletes in accordance with the minute schedule of starts (set by the event organizer). It happened that one athlete, starting three times in one session, filled out PANAS three times. There were also athletes starting only once in the session, they participated in one measurement with PANAS.

Each of the 31 athletes was therefore tested several times. We obtained in total 232 measurements of mood states (POMS) at the beginning of both sessions of the day (including 164 for men and 68 for women) and 362 measurements of emotional states just before start (PANAS) (248 for men, 114 for women). Due to a small number of respondents, the results of men and women were combined. Previous studies show that women and men – professional athletes do not differ in terms of mental traits important for this study (Olmedilla et al., 2018).

### Sports Results

Data on personal bests for a given distance and swimming style were obtained from respondents each time before the beginning of the race. The results achieved in the races were also recorded^1^. The time results were converted in order to compare the times achieved in different styles and distances. The personal record of an athlete was divided by the score obtained in the race and expressed as a percentage according to the formula: (previous personal best / result achieved in the race) × 100. A score of more than 100 means that the athlete achieved a better result than his or her personal best.

### Rank of Competition

Each time just before the race (start), after completing the PANAS the athlete evaluated the importance (rank) of a race on the 3-point scale: 1 – *low*, 2 – *medium*, 3 – *high*).

### Statistical Analysis

First, the normality of the investigated quantitative variables was checked using the Kolmogorov-Smirnov test. For most of the variables, distributions different from the Gaussian distribution were noted. For this reason, the Kruskal-Wallis test was used to determine the significance of intergroup differences. As the skewness values of the distributions fell within the range of +/− 2, it was considered that regression analysis could be performed. A stepwise regression analysis was used to determine the affective predictors of sports results. The sports result was the dependent variable. As factors, the indices of 7 moods (anger, confusion, depression, anxiety, fatigue, vigor, kindness) were entered into the equation. Regression analyzes were performed separately for races of low, medium and high subjective rank. A regression analysis was also carried out for all races, additionally introducing the rank of race as a factor. Then, analogous regression analyzes were performed with two emotional states (positive-negative) as factors.

## Results

The level of stress did not vary significantly depending on the subjective rank of the race (Table 1). The swimmers’ mood was the worst on the day of the race of low subjective rank (low perceived importance). Respondents revealed the highest level of negative states and a fairly low level of vigor. The highest level of vigor and the lowest level of four negative mood states were recorded on the day of the high-ranked race (high perceived importance).

**TABLE 1:** Stress levels and mood depending on the subjective rank of the race.

**Rank of race**	**Low rank**		**Medium rank**		**High rank**		**Kruskal-Wallis**	
	**(*n* = 30)**		**(*n* = 86)**		**(*n* = 126)**		**test**	
**Variable**	***M***	**SD**	***M***	**SD**	***M***	**SD**	**chi ^2^**	***p***
Stress	16.50	5.704	16.15	6.276	14.61	7.122	3.927	0.140
Anger	14.77	5.418	13.12	8.215	11.4	8.287	6.826	0.033
Confusion	11.70	4.268	9.99	4.423	8.80	4.728	11.345	0.003
Depression	19.57	9.111	16.15	12.02	13.63	10.184	10.2	0.006
Fatigue	18.70	4.268	13.99	6.762	10.09	6.715	49.394	<0.001
Tension	14.07	5.47	12.94	6.228	13.16	5.885	1.270	0.53
Vigor	13.43	4.321	15.26	5.129	17.42	6.138	11.087	<0.001
Kindness	17.07	5.948	17.56	4.991	17.13	4.733	1.114	0.573

In the case of emotional states prior to the start of the race, the differences were not so clear (Table 2). The level of positive emotions and negative emotions were significantly different depending on the subjective rank of the race. The level of both positive and negative emotions before the race of the high rank was higher than before the race with lower subjective rank (medium and low).

**TABLE 2:** The emotional states before the race depending on the rank of the race.

**Rank of race**	**low rank**		**medium rank**		**high rank**		**Kruskal-Wallis**	
	**(*n* = 52)**		**(*n* = 146)**		**(*n* = 164)**		**test**	
**Variable**	***M***	**SD**	***M***	**SD**	***M***	**SD**	**chi ^2^**	***p***
Positive emotions	23.06	9.918	27.65	8.431	32.68	7.582	51.233	<0.001
Negative emotions	14.21	5.263	15.11	5.991	16.02	5.399	11.552	0.003

The sports results obtained at the competition of *low* (M ± 98.15, SD ± 2.765), *medium* (M ± 98.93, SD ± 3.244) and *high* (M ± 99.89, SD ± 1.969) subjective rank were also compared. Their variability was found to be statistically significant (χ^2^ = 30.469, *p* < 0.001). The swimmers had better results in races which they rated as the most important, whereas lower scores were observed in less important races.

Stepwise regression analysis was carried out to determine whether the affective states are capable of predicting sports results. First, the predictors were searched for in terms of mood states, separately for the results obtained in low, medium and high-ranked competitions (Table 3).

**TABLE 3:** Mood states as predictors of sports result.

**Rank of race**	**Predictor**	**β**	***R*^2^**	***F***	***p***
Low	fatigue	−0.470	0.193	7.676	0.010
Medium	depression	−0.558	0.107	5.843	0.04
High	confusion	0.370	0.047	7.116	0.009
	depression	−0.235			
All races	depression	−0.265	0.133	18.926	<0.001
	rank	0.214			

In the case of low-ranked races, sports results can be predicted at almost 20% based on the level of fatigue, which is a negative predictor. Two predictors of the results were identified in middle-ranked races: depression and confusion. In the case of high-ranked races, only depression turned out to be a predictor. Mood states allowed for predicting sport performance during competitions that were assessed the athletes as important. Then, additional factor of a subjective rank of the race was added. Two predictors were revealed: depression (negative predictor) and the rank of the race (positive predictor).

It was also verified whether the emotional state just before the race allows for predicting the results obtained in a given race. The results of the race assessed as medium important and important can be predicted in about 5% based on a positive emotional state.

Two predictors were determined after the introduction of an additional factor of a subjective rank of the race. A positive emotional state and the rank of the race allow for predicting the sport result in about 13%. High results should be expected in high-rank races with swimmers experiencing strong positive pre-competition states measured just before the race (Table 4).

**TABLE 4:** Emotional states as predictors of a sports result.

**Rank of race**	**Predictor**	**β**	***R*^2^**	***F***	***p***
Low	–				
Medium	positive emotional state	0.252	0.057	9.281	0.03
High	positive emotional state	0.230	0.047	8.921	0.003
All starts	positive emotional state	0.249	0.133	18.926	<0.001
	rank	0.136			

## Discussion

The results of the study confirm our presumption that the subjective rank of competitions differentiates the swimmers’ mood. The mood was the best on the day of the high-rank races. On that day, athletes experienced the weakest negative mood states: fatigue, confusion, depression and anger. The level of vigor was the highest at the time. In other studies of swimmers, fatigue by the big amount of training caused a decrease in vigor (Faude et al., 2008). Especially big differences were observed in the level of fatigue: it was much lower before high-ranked races. The results obtained in our study do not question the findings of other researchers (Torres-Luque et al., 2013) concerning the importance of objectively determined rank of competitions (Gracz and Sankowski, 2007).

Swimmers experienced similar intensity of stress before races, regardless of their perceived importance (subjective rank). Differences in the levels of stress in three different conditions were not statistically significant.

Only negative moods were significant predictors of sports results, regardless of the subjective rank of race. The level of depression was a significant negative predictor of sports results obtained during medium- and high-rank races. The importance of this predictor was also confirmed in the analysis for all races. It can therefore be assumed that this mood is a particularly important factor determining the sports result. The relationship between depression and the appraisal of a difficult situation is carried in two directions. On the one hand, depression is likely to affect how an athlete perceives the situation and interprets his or her experiences (Wolf et al., 2015; Sahin et al., 2017). On the other hand, a difficult situation can make the athlete feel depressed.

In our swimmers, the level of emotions before the race, both positive and negative, was the highest in races of the high subjective rank. This confirms the findings of Gracz and Sankowski (2007) that the strength of the emotional experience of athletes depends on the rank of the competition: the higher the rank, the stronger the emotional excitation (Fernández et al., 2020). In similar study it turned out that strong negative emotions do not prevent athletes from achieving high sports results. The highest-ranked swimmers felt the strongest positive and negative emotions during the competitions, simultaneously achieving the highest sports results (Samełko et al., 2018). This confirms the observation of other researchers that both positive and negative emotions release energy, which has a positive impact on sport performance (Martinent et al., 2013). Feeling positive affective states may require more attention from athletes than experiencing negative states (Vast and Young, 2010).

Our results suggest that sport performance of swimmers, regardless of the subjective rank of the sporting event, can be predicted based on negative moods on the day of the competition. It is more likely that mood states, especially depression, affect sports results by modifying the cognitive appraisal of the starting situation and expectations of the result. It is worth noting at this point that their predictive power decreased with the rank of the competition. In the case of competitions subjectively assessed as the most important, the significance of mood as the predictor of sports result measured by the coefficient of determination was the lowest. Furthermore, the positive emotional state felt just before the race contributed to the mobilization of energy, which allowed for faster covering of the distance, but only when the athlete assigned a moderate or high rank to the race.

The study has limitations. The survey was conducted in a small group of athletes in one sport, which significantly reduces the possibilities for generalization of results. The only cognitive factor considered was a subjective assessment of the rank of the race. The authors are aware that the POMS tool is a quite old questionnaire. However, currently conducted research on athletes show justification for using this tool (Adrade et al., 2013; Rice et al., 2017).

## Final Considerations

Our results suggest that the pre-competitive mood and the emotions are related to performance, which is reflected in other studies (Patrícia et al., 2019). The subjective rank of the race may be a factor modifying the relationship between affective states and results. It seems appropriate to take measures aimed at increasing the ability of athletes to self-regulate emotionally (Doron and Martinent, 2017). Emotions should be an important area of mental preparation (Samełko et al., 2018). This allows for increasing the effectiveness of athletes also in stressful situations (Gross and Thompson, 2007; Church et al., 2017).

## Practical Implications

Based on the results obtained, the following guidelines can be formulated for the coaches and athletes:

1. Increased knowledge of coaches about how the athlete subjectively evaluates the importance of a sporting event may favor the optimization of athlete’s preparation for the competition;

2. Athletes should develop their affective awareness to cope better with the performance situation;

3. Coaches should educate themselves in recognizing the affective states of athletes for more effective training and performance during the competition;

4. The objective and subjective importance / rank of the competition should be taken into account by the coach and the athlete in the period of setting goals for the season.

## Data Availability Statement

The datasets presented in this article are not readily available because data appear in the doctoral dissertation. The entire dissertation is available for viewing at the library in Józef Piłsudski University of Physical Education. Requests to access the datasets should be directed to Violetta Perzyñska, biblioteka@awf.edu.pl.

## Ethics Statement

The studies involving human participants were reviewed and approved by the Senate Research Ethics Committee (Józef Piłsudski University of Physical Education in Warsaw). Written informed consent to participate in this study was provided by the participants’ legal guardian/next of kin.

## Author Contributions

AS data collection, research organization and project, and description of the results. MG research methodology, statistical analysis, and description of the results. AK collecting literature and manuscript proofreading. All authors contributed to the article and approved the submitted version.

## Conflict of Interest

The authors declare that the research was conducted in the absence of any commercial or financial relationships that could be construed as a potential conflict of interest.

## Footnotes

1 The results were publicly available online on the (Megatiming, 2018) website.
